# Silencing of MEG3 attenuated the role of lipopolysaccharides by modulating the miR-93-5p/PTEN pathway in Leydig cells

**DOI:** 10.1186/s12958-021-00712-5

**Published:** 2021-02-27

**Authors:** Xu Zhou, Jingliang He, Jinbo Chen, Yu Cui, Zhenyu Ou, Xiongbing Zu, Nenghui Liu

**Affiliations:** 1 Reproductive Medicine Center, Xiangya Hospital, Central South University, 87 Xiangya Road, Changsha, 410008 Hunan China; 2 Department of Urology, Xiangya Hospital, Central South University, 87 Xiangya Road, Changsha, 410008 Hunan China

**Keywords:** Orchitis, Leydig cell, LPS, MEG3, ceRNA

## Abstract

**Background:**

Leydig cells reflect the activation of inflammation, decrease of androgen production, inhibition of cell growth and promotion of cell apoptosis under orchitis. Maternally expressed gene 3 (MEG3) exerts a crucial role in various human diseases, but under orchitis, the role and underlying molecular mechanism of MEG3 in Leydig cells remain unclear.

**Methods:**

Lipofectamine 2000 was used for the cell transfections. qPCR and western blots assay were applied to assess the gene expression. ELISA assay was used to measure the TNFα, IL6 and testosterone secretion. CCK8 and EdU assay was employ to test the cell viability and proliferation respectively. Luciferase reporter and RIP assay were introduced to detect the binding of miR-93-5p with MEG3 and PTEN.

**Results:**

Lipopolysaccharides (LPS) induced TNFα and IL6 secretion, lowered testosterone production, inhibited cell viability and proliferation, and induced cell apoptosis in Leydig cells. MEG3 was upregulated in Leydig cells treated with LPS and that knockdown of MEG3 inhibited the role of LPS in Leydig cells. MEG3 absorbed miR-93-5p and that suppression of miR-93-5p restored the role of silenced MEG3 in Leydig cells under LPS treatment. miR-93-5p inhibited PTEN expression and that over-expressed PTEN alleviated the effect of miR-93-5p in Leydig cells treated with LPS. LPS activated the MEG3/miR-93-5p/PTEN signalling pathway in Leydig cells.

**Conclusions:**

This study revealed that MEG3 serves as a molecular sponge to absorb miR-93-5p, thus leading to elevation of PTEN expression in Leydig cells under LPS treatment, offering a theoretical basis on which to establish potential new treatment strategies for orchitis.

## Introduction

As a dominant cause of infertility, orchitis is defined as inflammation of one or both testicles [1]. The causes of orchitis include infections, trauma, and tumours, and the precise incidence of orchitis is also well known [2]. As male-specific cells, testicular Leydig cells perform the main function of synthesis and secretion of male hormones, including progesterone and testosterone, which promote spermatogenesis and male reproductive organ development and maintain secondary sexual characteristics and sexual function [3]. As an immune-inducing factor, bacterial lipopolysaccharides (LPS) can induce cell inflammation, inhibit cell proliferation and induce cell apoptosis [4]. LPS induces lipid peroxidation and apoptosis, decreases secretion of testosterone, disturbs spermatogenesis production and disrupts spermatogenesis in the testes [5, 6]. Therefore, understanding the role and the underlying mechanism of LPS in growth and testosterone production in Leydig cells might aid in development of innovative strategies for the treatment of orchitis.

Increasing evidence shows that less than 2% of the active genome is transcribed to protein-coding genes, indicating that most transcripts are non-coding RNAs (ncRNAs) [7]. NcRNAs were roughly classified into small ncRNAs with a length of less than 200 nucleotides and long ncRNAs (lncRNAs) with a length greater than 200 nucleotides [8, 9]. Although once considered “transcriptional noise”, lncRNAs play an important role in a variety of cellular progresses, including chromatin remodelling, cell proliferation and cycling, cell death, metastasis, development and tumourigenesis via regulation of genes at the transcriptional, post-transcriptional and translational levels [10–13].

LncRNAs exert influence in a great variety of human disease, including tumours and cancer, nervous system-related diseases, urogenital-related system diseases, cardiovascular-related diseases, gastrointestinal system-related diseases, embryonic development-related diseases and immune system-related diseases [14–16]. Sofia Boeg Winge and colleagues screened differential expressed lncRNAs in fixed paraffin-embedded testicular tissue samples and displayed the disturbance during differentiation of Leydig and Sertoli cells [17]. However, no literature is available to further elucidate the role and underlying mechanism of lncRNAs in the Sertoli cells of orchitis.

Located in chromosome 14q32, lncRNA maternally expressed gene 3 (MEG3) acts as a tumour suppressor and is involved in physiological and pathological progression of various human diseases [14, 18]. MEG3 has been found to play a crucial role in various inflammation-related diseases. Zhaolin Wang and colleagues reported that knockdown of MEG3 attenuated LPS-induced inflammatory injury in ATDC5 cells [19]. However, there is a lack of focus in the literature on the functional roles and underlying mechanism of MEG3 in Leydig cells under orchitis.

Therefore, this study aimed to investigate the role and further dissect the underlying molecular mechanism of MEG3 in Leydig cells under orchitis. This study successfully elucidated the role and underlying mechanism of MEG3 in Leydig cells under LPS and revealed a novel regulatory signalling pathway that offers a certain degree of potential for orchitis treatment.

## Materials and methods

### Cell culture, treatment and transfection

Human Leydig cells were purchased from ScienCell Research Laboratories (ScienCell, San Diego, California, USA) and routinely conserved in our labs and cultured in Dulbecco’s modified Eagle medium (DMEM; GIBCO, NY, USA) supplemented with 10% foetal bovine serum (FBS; Invitrogen, CA, USA), 10 U/mL penicillin and 10 μg/mL streptomycin. Cells were maintained in a 37 °C/5% CO_2_ humidified incubator. LPS (Sigma-Aldrich, MO, USA) was dissolved in the medium. The cells were treated with LPS (Sigma-Aldrich, MO, USA) at different concentrations for approximately 48 h. Transfection of small interfering RNA (siRNAs), miR-93 inhibitor, miR-93 mimics and control small RNAs (GenePharma, Shanghai, China) was performed using Lipofectamine 2000 (Thermo Fisher Scientific, CN, USA) over a period of approximately 48 h.

### RNA isolation and real-time quantitative PCR

Trizol reagent (Junxin, Suzhou, China) was introduced to extract the total RNA. A NanoDrop 2000 (Thermo Fisher) instrument was used to measure the concentration and purity of total RNA. A reverse transcription kit (Takara, Dalian, China) was applied to synthesize the first-strand cDNA. The 2 × SYBR Green qPCR Mix (Junxin, Suzhou, China) was selected to perform qPCR analysis. The expression levels for lncRNA and mRNA were normalized to β-actin expression, and the expression levels for miRNAs were relative to the U6 expression. The 2^−ΔΔCt^ method and arbitrary units were introduced to calculate the relative fold change. The primers used in this study are listed in Table 1.

**Table 1:** Primers used in this study

Gene	primer	Sequence
MEG3	Forward	5′- GGCAGGATCTGGCATAGAGG − 3’
	Reverse	5′- CGAGTCAGGAAGCAGTGGGTT-3’
PTEN	Forward	5′- ACCCACACGACGGGAAGACA − 3’
	Reverse	5′- CTGTTTGTGGAAGAACTCTACTTTGATATCAC − 3’
β-actin	Forward	5′- CATTCCAAATATGAGATGCGTTGT − 3’
	Reverse	5′- TGTGGACTTGGGAGAGGACT − 3’
miR-93-5p	Forward	5′- GTCACAAAGUGCUGUUCGUGC-3’
	Reverse Transcription	5′-GTCGTATCCAGTGCAGGGTCCGAGGTATTCGCACTGGATACGACCTACCTG − 3’
U6	Forward	5′- CTCGCTTCGGCAGCACA-3’
	Reverse	5′- AACGCTTCACGAATTTGCGT-3’

### Western blot analysis

The radio immunoprecipitation assay (RIPA) (Takara, Dalian, China) was introduced to isolate the protein. The BCA protein detection kit (Junxin, Suzhou, China) was applied to measure the concentration of protein. The proteins (30 μg in each lane) were separated via sodium dodecyl sulfate-polyacrylamide gel electrophoresis (SDS-PAGE) and transferred onto polyvinylidene fluoride (PVDF) membranes (Millipore, USA). The membranes were blocked in 5% fat-free milk and exposed to different primary antibodies at 4 °C overnight. The membranes were incubated with horseradish peroxidase-conjugated IgG. The immunoreactive protein bands were detected with a chemiluminescence (ECL) reagent (Junxin, Suzhou, China) and the Bio-Rad ChemiDocTM XRS system. β-Actin was measured as a loading control. The antibodies used in this study are listed below: The anti-PTEN antibody (Ab267787, Abcam, Cambridge, British), anti-Bcl-2 antibody (Ab32124, Abcam, Cambridge, British), anti-Bax antibody (Ab182734, Abcam, Cambridge, British), anti-β-Actin (Ab207604, Abcam, Cambridge, British) and Goat Anti-Rabbit IgG H&L (HRP) (Ab 6721, Ab207604, Abcam, Cambridge, British) were used according to the protocol.

### Cell counting kit-8 (CCK-8) assay

Cell viability was tested using the CCK-8 assay (Junxin, Suzhou, China). In brief, cells were plated in a 96-well cell culture plate at a concentration of 4 × 10^3^ cells per well (3 replicates for each experimental condition). After different treatments, 10 μl CCK-8 reagent was added to the cells in each well and incubated for 2 h in a 5% CO_2_ humidified incubator at 37 °C. The absorbance of the cells at 450 nm was detected using a microplate reader (Bio-Rad, Hercules, USA).

### 5-ethynyl-2′-deoxyuridine (EdU) incorporation assay

The cell proliferation potential was assessed using an EdU kit (Junxin, Suzhou, China). In brief, cells were plated in a 48-well cell culture plate at a concentration of 5 × 10^4^ cells per well (3 replicates for each experimental condition). After different treatments, 200 μl EdU reagent was added to the cells in each well and incubated for 2 h in a 5% CO_2_ humidified incubator at 37 °C. The EdU analysis was performed according to the manufacturer’s instructions. The images were captured using a fluorescence microscope (Olympus, Tokyo, Japan).

### Construction of reporter vectors and luciferase reporter assay

The fragments of MEG3 and PTEN containing the putative miR-93 binding site were amplified and inserted into the psiCheck2 Dual-Luciferase miRNA Target Expression Vector (Promega, Madison, WI, USA) to construct the reporter vectors MEG3-wild-type (MEG3-wt) and PTEN-wild-type (PTEN-wt), respectively. The psiCheck2 vectors cloned with the fragments of MEG3 and PTEN containing the mutant miR-93 binding site were referred to as MEG3-mutant-type (MEG3-mut) and PTEN-mutant-type (PTEN-mut), respectively. These reporters were co-transfected into HEK293 cells with miR-93 mimics using Lipofectamine 2000. The luciferase activities were measured with a Dual-Luciferase® Reporter Assay System Protocol (Promega).

### RNA immunoprecipitation (RIP) assay

The RIP analysis was performed with the RIP kit (Millipore, MA USA) and anti-Argonaute 2 (Ago2) antibodies (Abcam, USA) according to the protocol. In brief, the cells were lysed in RNA lysis buffer, and the cell lysate was incubated with Protein A/G beads (Biolinkedin, Shanghai, China) and anti-Argonaute 2 (Ago2) antibodies (Abcam, USA) at 4 °C overnight. IgG was added as a negative control. The immunoprecipitated RNA was isolated and reverse transcribed, and qPCR analysis was introduced to measure the enrichment of MEG3 and miR-93.

### Enzyme-linked immunosorbent assay (ELISA)

After different treatments, the culture supernatants were harvested, and the concentrations of the inflammatory cytokines of interleukin-6 (IL6), tumour necrosis factor alpha (TNFα) and testosterone were tested using ELISA kits (Thermo Fisher Scientific, Waltham, USA) according to the manufacturer’s protocol.

### Statistical analysis

All experiments were repeated three times. Data are expressed as the mean ± standard deviation (SD). Graphpad Prism 5 software (GraphPad, San Diego, CA) was used to perform the statistical analysis. The *p*-values of two independent comparisons were compared using Student’s t-test, and multi-group comparisons were expressed using one-way ANOVA. A p-value of < 0.05 indicated a significant difference.

## Results

### LPS induced dysfunction and inhibited cell growth in Leydig cells

**Fig. 1:**
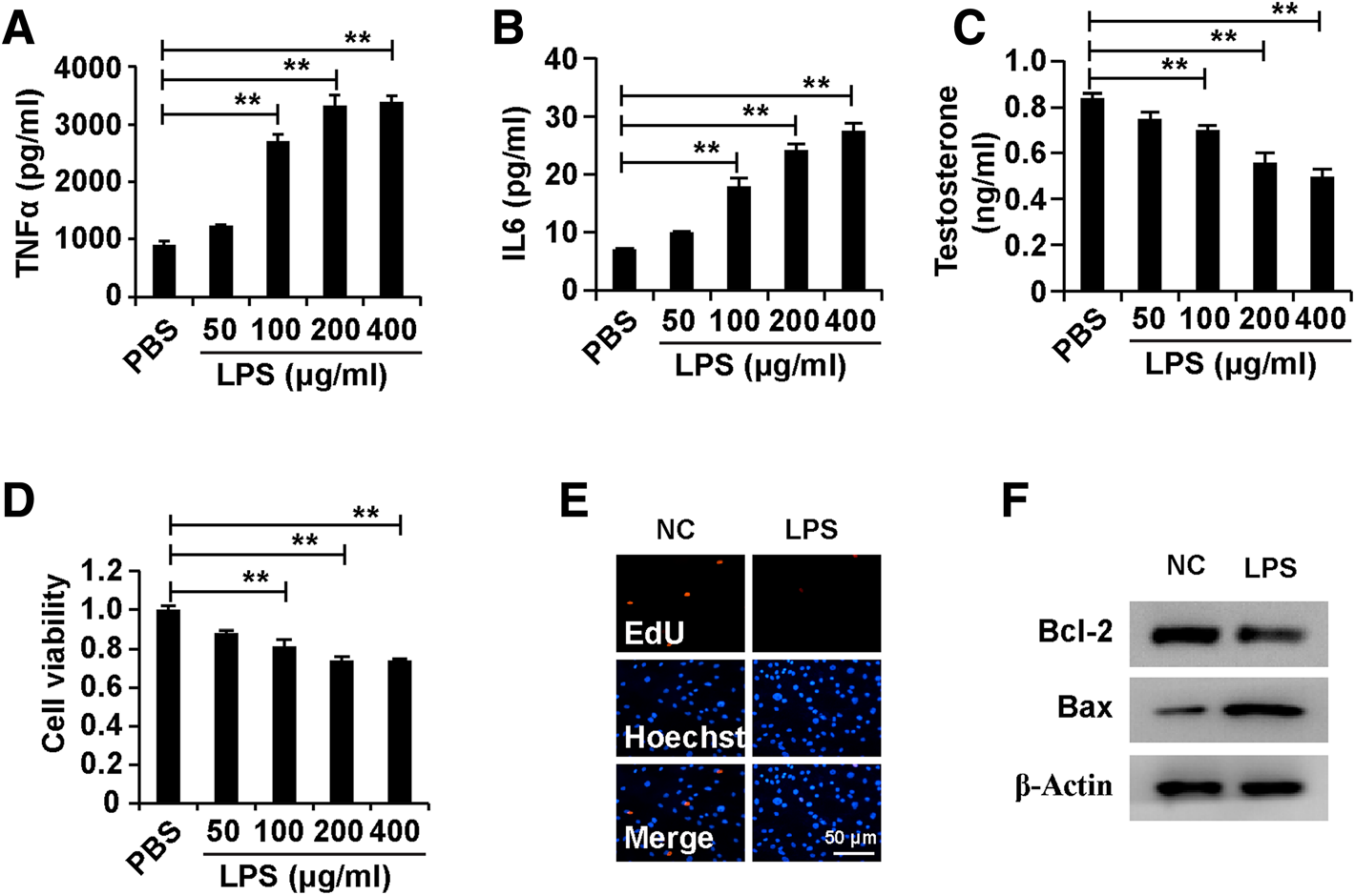
LPS induced dysfunction and inhibited cell growth in Leydig cells. (A) ELISA analysis of secretion of TNFα and IL6 in Leydig cells. (B) ELISA analysis of testosterone production in Leydig cells. (C) CCK8 analysis of cell viability in Leydig cells. (D) EdU analysis of cell proliferation in Leydig cells. (E) Western blot analysis of cell apoptosis in Leydig cells. For A-C, Leydig cells were treated with different concentrations of LPS (0, 50, 100, 200, 400 μg/ml) for 48 h. For D and E, Leydig cells were treated with LPS at a dose of 200 μg/ml for 48 h. β-Actin was measured as a loading control for western blot analysis. “**” represents *p* < 0.01, which indicates significantly statistic differences between two groups in Student’s t-test

To determine the role of LPS in Leydig cells, we treated Leydig cells with different concentrations of LPS (0, 50, 100, 200, and 400 μg/ml) for 48 h. ELISA analysis demonstrated that LPS induced the secretion of TNFα and IL6 in Leydig cells (Fig. 1a and b). Further research demonstrated that LPS inhibited the testosterone production in Leydig cells (Fig. 1c). The results of CCK8 analysis showed that LPS reduced the cell viability of Leydig cells (Fig. 1d). The results of EdU analysis showed that LPS decreased the cell proliferation of Leydig cells (Fig. 1e). Ultimately, western blot analysis revealed that LPS induced cell apoptosis, decreased Bcl-2 and increased Bax in Leydig cells (Fig. 1f). In summary, these results suggested that LPS induced dysfunction in Leydig cells.

### Silencing of MEG3 attenuated the role of LPS in Leydig cells

**Fig. 2:**
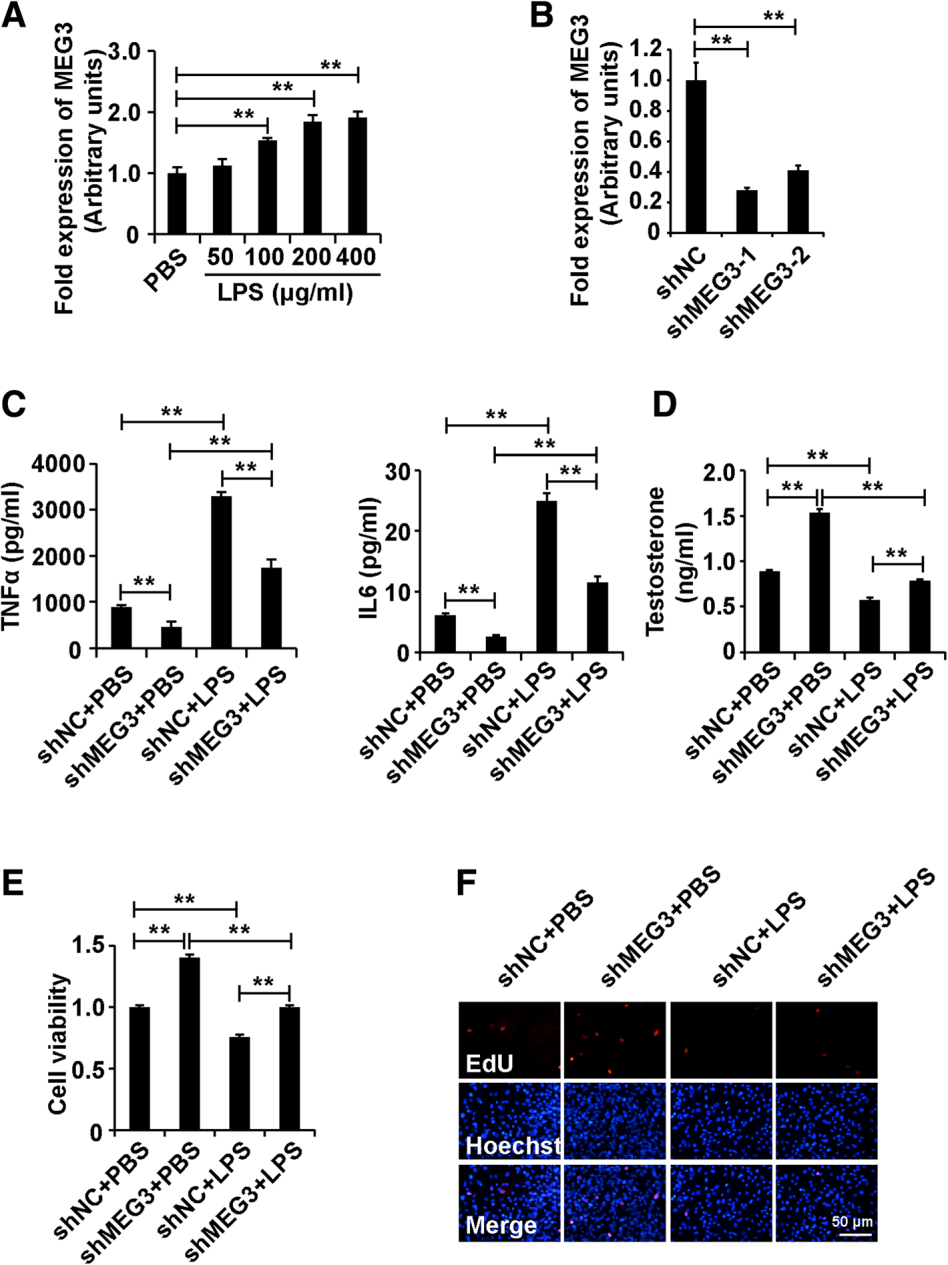
Silencing of MEG3 attenuated the role of LPS in Leydig cells. (A) qPCR analysis of MEG3 expression in Leydig cells treated with different concentrations of LPS (0, 50, 100, 200, 400 μg/ml) for 48 h. (B) qPCR analysis of MEG3 expression in Leydig cells transfected with siMEG3s and siNC for 48 h. (C) ELISA analysis of secretion of TNFα and IL6 in Leydig cells. (D) ELISA analysis of testosterone production in Leydig cells. (E) CCK8 analysis of cell viability in Leydig cells. (F) EdU analysis of cell proliferation in Leydig cells. For C-F, Leydig cells were divided into four groups: shNC, shMEG3, shNC+LPS and shMEG3 + LPS. β-Actin was measured as an internal control for qPCR analysis. “**” represents *p* < 0.01, which indicates statistically significant differences between two groups in Student’s t-test

To investigate whether MEG3 was involved in the role of LPS in Leydig cells, we measured the expression of MEG3 in Leydig cells treated with different concentrations of LPS. The results of qPCR analysis demonstrated that LPS increased the expression level of MEG3 in Leydig cells (Fig. 2a). This observation indicated that MEG3 participated in the role of LPS in Leydig cells.

To test whether MEG3 affected the role of LPS in Leydig cells, we first designed and synthesized short-hair RNA specific against MEG3 (shMEG3s) and control short-hair RNA (shNC). We transfected shMEGs and shNC into Leydig cells for 48 h. The results of qPCR analysis demonstrated that both shMEG3s could efficiently downregulate the expression of MEG3 in Leydig cells, and shMEG3–1 was chosen for further experiments (Fig. 2b).

We divided the cells into four groups: shNC, shMEG3, shNC+LPS and shMEG3 + LPS. The ELISA analysis demonstrated that silencing of MEG3 decreased the secretion of TNFα and IL6 and inhibited LPS-induced promotion of TNFα and IL6 secretion in Leydig cells (Fig. 2c). Furthermore, knockdown of MEG3 elevated testosterone production and lowered LPS-induced testosterone production in Leydig cells (Fig. 2d). In addition, suppression of MEG3 enhanced the cell viability and proliferation and attenuated the LPS-induced inhibition of cell viability and proliferation in Leydig cells (Fig. 2e and f).

### MEG3 absorbed miR-93-5p and miR-93-5p alleviated the role of LPS in Leydig cells

**Fig. 3:**
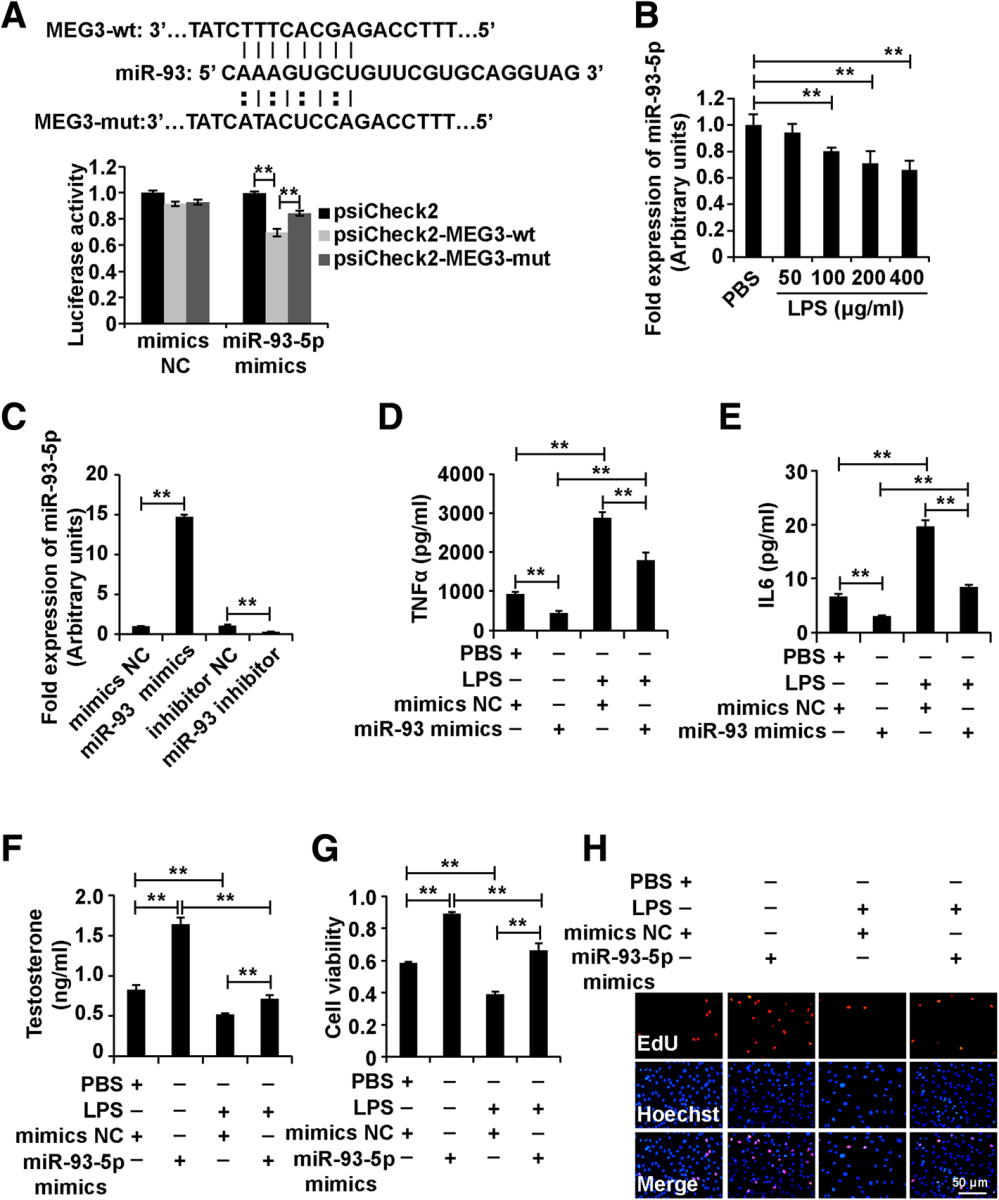
MEG3 absorbed miR-93-5p and miR-93-5p alleviated the role of LPS in Leydig cells. (A and B) Luciferase reporter system analysis of the interaction between MEG3 and miR-93-5p. (C) qPCR analysis of miR-93-5p expression in Leydig cells treated with different concentrations of LPS (0, 50, 100, 200, 400 μg/ml) for 48 h. (D) qPCR analysis of miR-93-5p expression in Leydig cells transfected with miR-93-5p mimics, mimics NC, miR-93-5p inhibitor and inhibitor NC for 48 h. (E) ELISA analysis of secretion of TNFα and IL6 in Leydig cells. (F) ELISA analysis of testosterone production in Leydig cells. (G) CCK8 analysis of cell viability in Leydig cells. (H) EdU analysis of cell proliferation in Leydig cells. For E-H, Leydig cells were divided into four groups: mimics NC, miR-93-5p mimics, mimics NC + LPS and miR-93-5p mimics+LPS. U6 was measured as an internal control for qPCR analysis. “**” represents *p* < 0.01, which indicates statistically significant differences between two groups in Student’s t-test

To dissect the molecular mechanism of MEG3 in LPS in Leydig cells, we used the miRNAs prediction online site to screen the candidate targets of MEG3 and found that miR-93-5p was a candidate of MEG3. We introduced the luciferase reporter system to confirm the interaction between MEG3 and miR-93-5p. First, we cloned MEG3 transcripts containing the predicted binding site of miR-93-5p and the mutant binding site of miR-93-5p into the luciferase reporter vector psiCheck2, referred to as psiCheck2-MEG3 wild type (psiCheck2-MEG3-wt) and psiCheck2-MEG3 mutant type (psiCheck2-MEG3-mut), respectively. We transfected psiCheck2-MEG3-wt and psiCheck2-MEG3-mut together with miR-93-5p mimics into 293 cells. At 48 h, the luciferase activity was measured, and the results demonstrated that miR-93-5p mimics inhibited the luciferase activity of psiCheck2-MEG3-wt, and the luciferase activity was restored in psiCheck2-MEG3-mut (Fig. 3a). Taken together, these results demonstrated that miR-93-5p could bind to the predicted binding site of miR-93-5p on MEG3 transcripts.

To investigate whether miR-93-5p affected the role of LPS on Leydig cells, we first measured the expression of miR-93-5p in Leydig cells treated with different concentrations of LPS, and the results of qPCR analysis demonstrated that LPS decreased the expression level of miR-93-5p in Leydig cells (Fig. 3b). This observation indicated that miR-93-5p participated in the role LPS in Leydig cells. We synthesized the miR-93-5p mimics, mimics NC, miR-93-5p inhibitor and inhibitor NC, and the results of qPCR analysis demonstrated that the miR-93-5p mimics significantly increased the miR-93-5p level and the miR-93-5p inhibitor significantly decreased that in Leydig cells (Fig. 3c).

In addition, we divided the cells into four groups: mimics NC, miR-93-5p mimics, mimics NC + LPS and miR-93-5p mimics+LPS. The ELISA analysis demonstrated that miR-93-5p decreased the secretion of TNFα and IL6 and inhibited the LPS-induced promotion of TNFα and IL6 secretion in Leydig cells (Fig. 3d and e). Furthermore, miR-93-5p increased the testosterone production and decreased the LPS-induced testosterone production of Leydig cells (Fig. 3f). In addition, miR-93-5p enhanced the cell viability and proliferation and attenuated the LPS-induced inhibition of cell viability and proliferation in Leydig cells (Fig. 3g and h). Taken together, these results showed that MEG3 absorbed miR-93-5p and that miR-93-5p alleviated the role of LPS on Leydig cells.

### Inhibition of miR-93-5p alleviated the role of silenced MEG3 in Leydig cells treated with LPS

**Fig. 4:**
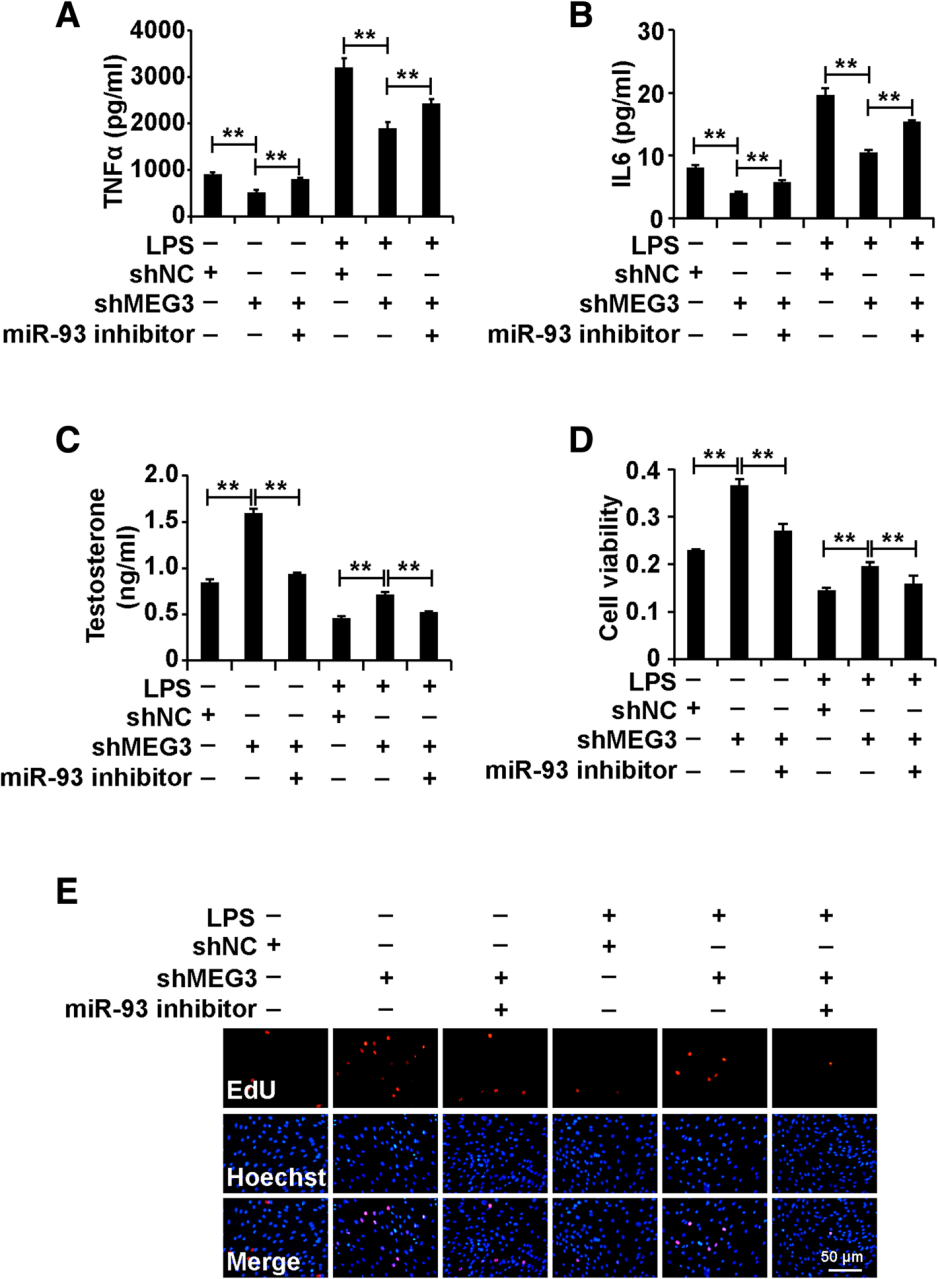
Inhibition of miR-93-5p alleviated the role of silenced MEG3 in Leydig cells treated with LPS. (A) ELISA analysis of secretion of TNFα and IL6 in Leydig cells. (B) ELISA analysis of testosterone production in Leydig cells. (C) CCK8 analysis of cell viability in Leydig cells. (D) EdU analysis of cell proliferation in Leydig cells. For A-D, Leydig cells were divided into six groups: shNC+inhibitor NC, shMEG3 + inhibitor NC, shMEG3+ miR-93-5p inhibitor, shNC+inhibitor NC + LPS, shMEG3 + inhibitor NC + LPS, and shMEG3+ miR-93-5p inhibitor+LPS. “**” represents p < 0.01, which indicates statistically significant differences between two groups in Student’s t-test

To investigate whether miR-93-5p affected the role of MEG3 in Leydig cells treated with LPS, we divided the cells into six groups: shNC+inhibitor NC, shMEG3 + inhibitor NC, shMEG3+ miR-93-5p inhibitor, shNC+inhibitor NC + LPS, shMEG3 + inhibitor NC + LPS, and shMEG3+ miR-93-5p inhibitor+LPS. The ELISA analysis demonstrated that miR-93-5p inhibitor restored the shMEG3-induced inhibition of TNFα and IL6 secretion of Leydig cells (Fig. 4a and b). Furthermore, the ELISA analysis demonstrated that miR-93-5p inhibitor alleviated the shMEG3-induced elevation of testosterone production of Leydig cells (Fig. 4c). In addition, miR-93-5p inhibitor attenuated the shMEG3-induced enhancement of cell viability and proliferation in Leydig cells (Fig. 4d and e). In summary, these results suggested that inhibition of miR-93-5p alleviated the role of silenced MEG3 in Leydig cells treated with LPS.

### miR-93-5p regulated PTEN expression in Leydig cells

**Fig. 5:**
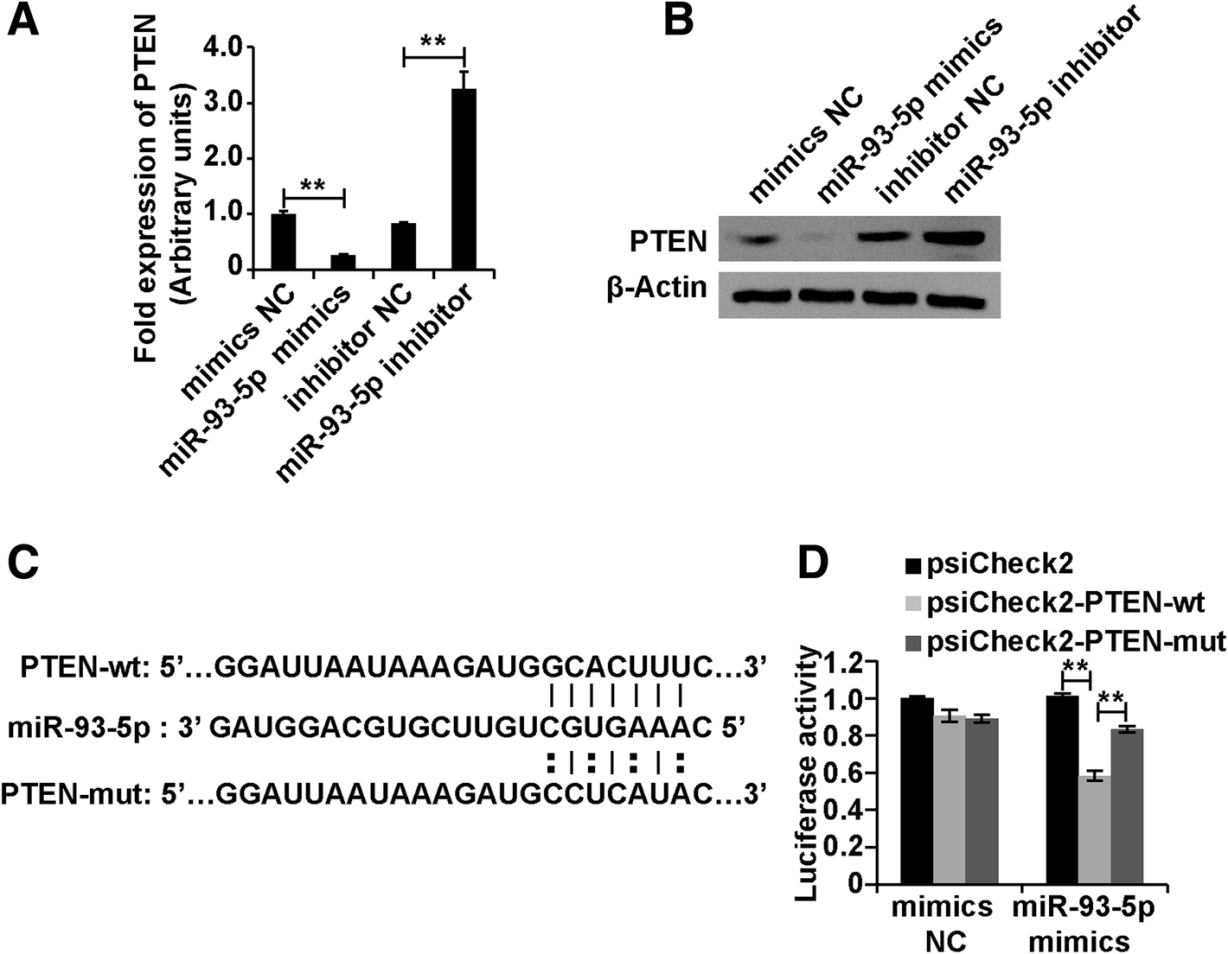
miR-93-5p regulated PTEN expression in Leydig cells. (A and B) qPCR and western blot analysis of PTEN expression in Leydig cells transfected with miR-93-5p mimics, mimics NC, miR-93-5p inhibitor and inhibitor NC for 48 h. (C and D) Luciferase reporter system analysis of the interaction between MEG3 and miR-93-5p. β-Actin was measured as a loading control for western blot analysis. “**” represents *p* < 0.01, which indicates statistically significant differences between two groups in Student’s t-test

To investigate the molecular mechanism of miR-93-5p in Leydig cells, we used the miRNAs prediction online site to screen the candidate target of miR-93-5p and found that PTEN was a candidate of miR-93-5p. To detect whether miR-93-5p could regulate the PTEN expression, we transfected the miR-93-5p mimics, mimics NC, miR-93-5p inhibitor and inhibitor NC into Leydig cells. The results of qPCR and western blots analysis showed that the miR-93-5p mimics inhibited and miR-93-5p inhibitor promoted the PTEN expression in Leydig cells (Fig. 5a and b). To further confirm the interaction between miR-93-5p and PTEN, we applied the luciferase reporter system as a test. First, we cloned the PTEN 3′ UTR containing the predicted binding site of miR-93-5p and the mutant binding site of miR-93-5p into the luciferase reporter vector psiCheck2, known as psiCheck2-PTEN wild type (psiCheck2-PTEN-wt) and psiCheck2-PTEN mutant type (psiCheck2-PTEN-mut), respectively. We transfected psiCheck2-PTEN-wt and psiCheck2-PTEN-mut together with miR-93-5p mimics into 293 cells. At 48 h, the luciferase activity was measured. The results demonstrated that miR-93-5p mimics inhibited the luciferase activity of psiCheck2-PTEN-wt, and the luciferase activity was restored in psiCheck2-PTEN-mut (Fig. 5c and d). Taken together, these results demonstrated that miR-93-5p inhibited PTEN expression in Leydig cells.

### Over-expression of PTEN attenuated the role of miR-93-5p mimics in Leydig cells treated with LPS

**Fig. 6:**
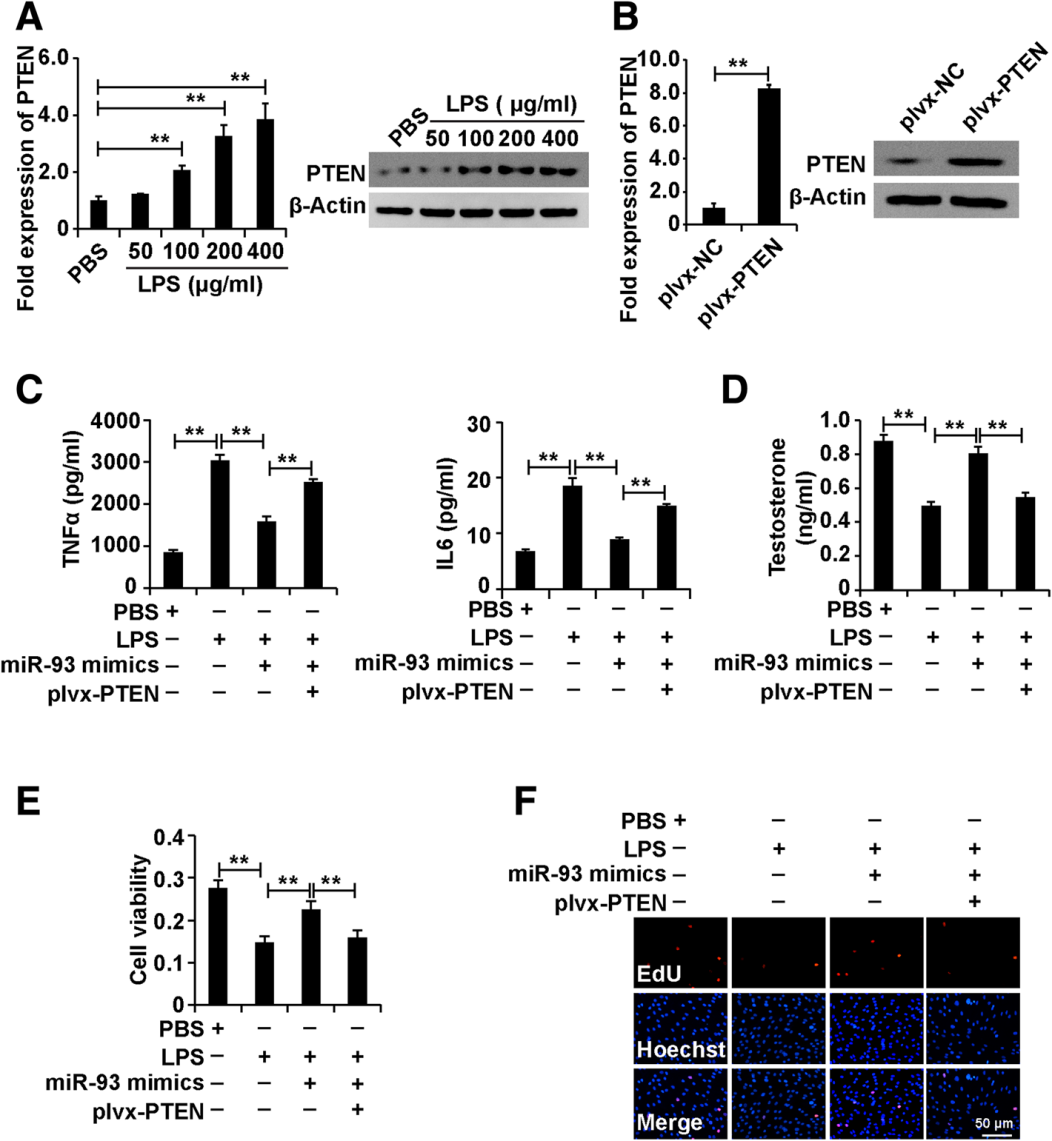
Over-expression of PTEN attenuated the role of miR-93-5p mimics in Leydig cells treated with LPS. (A) qPCR and western blot analysis of PTEN expression in Leydig cells treated with different concentrations of LPS (0, 50, 100, 200, 400 μg/ml) for 48 h. (B) qPCR and western blot analysis of PTEN expression in Leydig cells transfected with plvx-PTEN and plvx-NC for 48 h. (C) ELISA analysis of secretion of TNFα and IL6 in Leydig cells. (D) ELISA analysis of testosterone production in Leydig cells. (E) CCK8 analysis of cell viability in Leydig cells. (F) EdU analysis of cell proliferation in Leydig cells. For C-F, Leydig cells were divided into four groups: mimics NC, LPS + mimics NC, LPS+ miR-93-5p mimics and LPS + miR-93-5p mimics+plvx-PTEN. β-Actin was measured as an internal control for qPCR analysis. “**” represents p < 0.01, which indicates statistically significant differences between two groups in Student’s t-test

To investigate whether PTEN affected the role of miR-93-5p in Leydig cells treated with LPS, we first detected the PTEN expression in Leydig cells treated with different concentrations of LPS. The results of qPCR and western blot analyses showed that LPS elevated the expression level of PTEN in Leydig cells (Fig. 6a), indicating that PTEN was involved in the role of LPS in Leydig cells.

Furthermore, we constructed overexpression clones of PTEN based on the plvx vector (plvx-PTEN), and qPCR and western blot analyses revealed that overexpression clones of PTEN significantly elevated the expression level of PTEN in Leydig cells (Fig. 6b).

We divided the cells into four groups: mimics NC, LPS + mimics NC, LPS+ miR-93-5p mimics and LPS + miR-93-5p mimics+plvx-PTEN. The ELISA analysis demonstrated that plvx-PTEN restored miR-93-5p mimics-induced inhibition of TNFα and IL6 secretion of Leydig cells (Fig. 6c). Furthermore, the ELISA analysis demonstrated that plvx-PTEN alleviated miR-93-5p mimics -induced elevation of testosterone production in Leydig cells (Fig. 6d). In addition, plvx-PTEN attenuated miR-93-5p mimic-induced enhancement of cell viability and proliferation in Leydig cells (Fig. 6e and f). In summary, these observations revealed that upregulation of PTEN restored the role of miR-93-5p in Leydig cells treated with LPS.

### LPS activated the MEG3/miR-93-5p/PTEN signalling pathway in Leydig cells

**Fig. 7:**
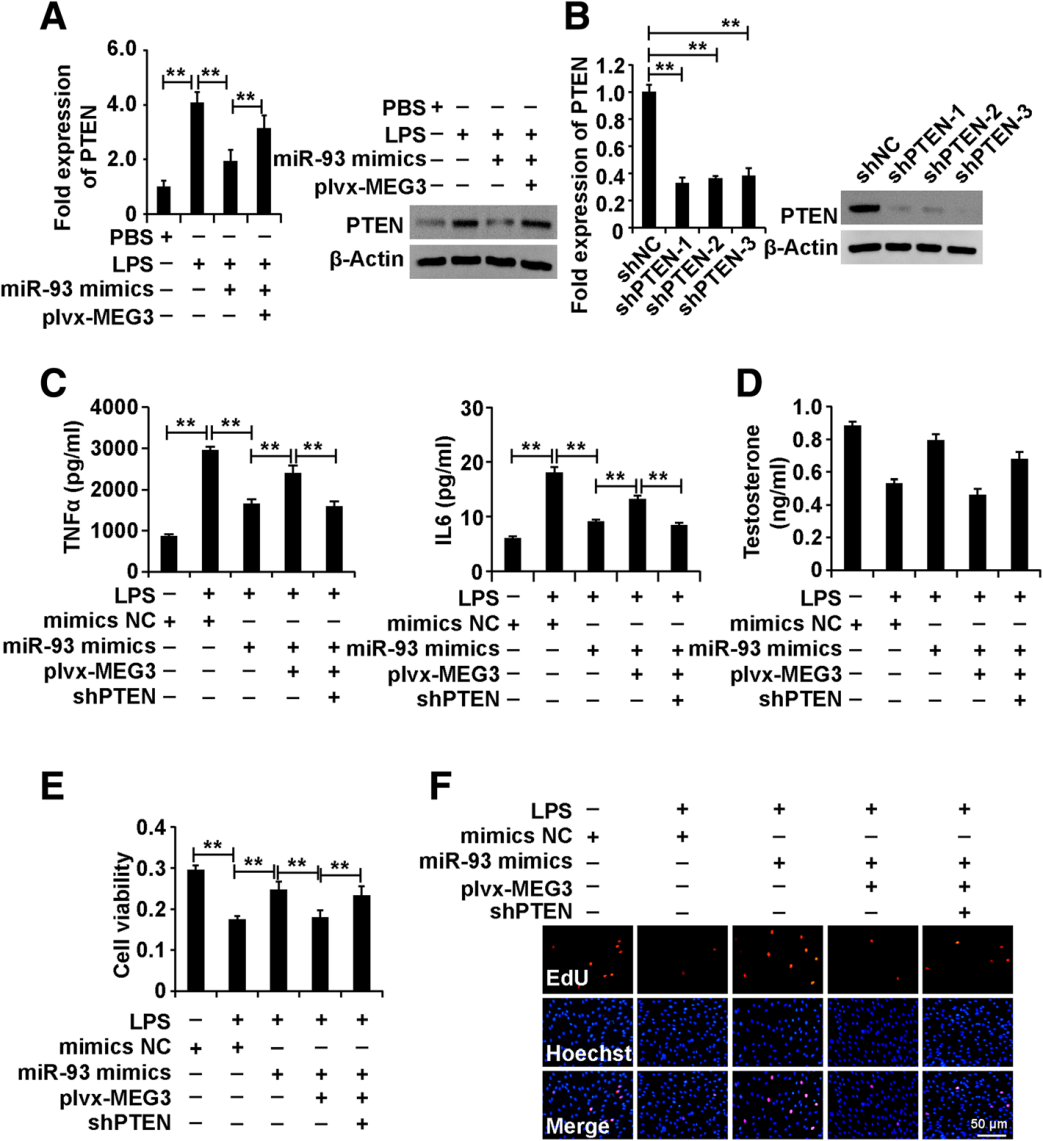
LPS activates the MEG3/miR-93-5p/PTEN signalling pathway in Leydig cells. (A) qPCR and western blot analysis of PTEN expression in Leydig cells divided into four groups: mimics NC, LPS + mimics NC, LPS+ miR-93-5p mimics and LPS + miR-93-5p mimics+plvx-MEG3. (B) qPCR and western blot analysis of PTEN expression in Leydig cells transfected with shPTENs and shNC for 48 h. (C) ELISA analysis of secretion of TNFα and IL6 in Leydig cells. (D) ELISA analysis of testosterone production in Leydig cells. (E) CCK8 analysis of cell viability in Leydig cells. (F) EdU analysis of cell proliferation in Leydig cells. For C-F, Leydig cells were divided into five groups: mimics NC, LPS + mimics NC, LPS+ miR-93-5p mimics, LPS + miR-93-5p mimics+plvx-MEG3 and LPS + miR-93-5p mimics+plvx-MEG3 + shPTEN. β-Actin was measured as an internal control for qPCR analysis. “**” represents p < 0.01, which indicates statistically significant differences between two groups in Student’s t-test

To determine whether MEG3 exerted its effect by regulating the miR-93-5p/PTEN pathway in Leydig cells treated with LPS, we first measured whether PTEN was regulated by MEG3/miR-93-5p pathway in Leydig cells treated with LPS. The results demonstrated that inhibition of LPS-induced PTEN by miR-93-5p mimics was restored by overexpressed MEG3 (Fig. 7a). These results demonstrated that MEG3 could alleviate the repression of PTEN by miR-93-5p in Leydig cells treated with LPS.

We designed and synthesized short-hair RNA specific against PTEN (shPTENs) and control short-hair RNA (shNC). We transfected shPTENs and shNC into the Leydig cells for 48 h. The results of qPCR analysis demonstrated that all three shPTENs could efficiently inhibit PTEN expression in Leydig cells, and shPTEN-3 was chosen for further experiments (Fig. 7b).

To determine the role of the MEG3/miR-93-5p/PTEN signalling pathway in Leydig cells treated with LPS, we divided the cells into five groups: mimics NC, LPS + mimics NC, LPS+ miR-93-5p mimics, LPS + miR-93-5p mimics+plvx-MEG3 and LPS + miR-93-5p mimics+plvx-MEG3 + shPTEN. The ELISA, CCK8 and EdU analyses demonstrated that any disturbers of the MEG3/miR-93-5p/PTEN signalling pathway affected the role of LPS in Leydig cells (Fig. 7c-f). In summary, these resulted revealed that LPS played a role via activation of the MEG3/miR-93-5p/PTEN signalling pathway in Leydig cells.

## Discussion

Infertility is a heavy global public health threat that is estimated to affect one out of every twenty men [20]. The male factor, which contains exposure to hazardous chemicals and radiation, excessive alcohol consumption, tobacco smoking and other unhealthy lifestyle factors, accounts for more than 50% infertility due to decreased sperm quality [21]. In the testes, autoimmune orchitis, which is induced by systemic inflammation such as immunosuppression, inflammatory reactions and infection, leads to testicular dysfunction, germ cell apoptosis, and inhibition of Leydig cell steroidogenesis and spermatogenesis, thus resulting in male infertility [2, 22]. Therefore, immune homeostasis is essential for normal function of the testes. This study demonstrated that LPS induced an increase of TNFα and IL6 secretion and a decrease of testosterone secretion, inhibited cell growth and induced apoptosis in Leydig cells. Therefore, an urgent need exists to investigate the role and underlying mechanism of LPS in the cellular progression and function of Leydig cells.

LncRNAs are divided into three main categories: intronic lncRNA (mainly produced in the intron region of the coding gene) intergenic lncRNA (mainly produced in the middle region of the two coding genes) and antisense lncRNA (mainly produced in the antisense strand of the coding gene) [8, 23]. Recently, more numerous functional lncRNAs were identified in human diseases [14, 24]. Several studies revealed the lncRNAs involved in spermatogenesis, steroidogenesis and cell differentiation in the testes. One study measured the RNA profile of human testicular cells to identify the lncRNAs associated with spermatogenesis [25]. Another research study systematically screened the enrichment of long non-coding RNAs that affected the differentiation of Sertoli and Leydig cells via transcriptome analysis of adults with Klinefelter syndrome (KS; 47,XXY) [17]. Yui Satoh and colleagues revealed that Tesra, which is a novel testis-specific lncRNA, regulated the Prss42/Tessp 2 gene and thus affected mouse spermatogenesis [26]. This study demonstrated that MEG3, a lncRNA, was increased in a LPS-treated mice model and in Leydig cells. Furthermore, knockdown of MEG3 alleviated the role of LPS in inflammatory factors and androgen secretion and cell growth of Leydig cells.

Recently, several regulatory mechanisms of lncRNAs were identified: combined with chromatin, which affects chromatin remodelling and generally affectis gene transcription; combined with the 5’UTR of genes, which affects gene transcription initiation; combined with mRNA transcripts, which affects mRNA alternative splicing, transport, and translation efficiency; binding to protein, which affects the location, modification and activity of protein; as a precursor of miRNA and siRNA, which affects the amount and activity of miRNA; and as a molecular sponge binding miRNA, which affects the amount of free miRNA, thus affecting the expression level of miRNA downstream target genes and activity [10, 27, 28].

The competing endogenous RNAs (ceRNA) hypothesis has attracted increasing attention from scientists [29]. The ceRNA hypothesis defined as that, lncRNAs which behaves binding site of miRNA serves as a molecular sponge to absorb the miRNA and thus, lowers the free miRNAs levels in cytoplasm, resulting in the decrease of the binding between miRNAs and its target-mRNA, leading to the elevation of gene transcripts and proteins [29, 30]. MEG3 was reported to exert its function via the ceRNA hypothesis [31]. Ru Yang and colleagues demonstrated that silencing of MEG3 alleviated LPS-induced apoptosis by sponging miR-21 in renal tubular epithelial cells [32]. This study demonstrated that MEG3 affected the role of LPS by sponging miR-93-5p, thus modulating PTEN expression in human Leydig cells.

PTEN (phosphatase and tensin homologue deleted on chromosome ten) was first identified as a tumour suppressor gene and has an anti-growth function in a variety of cellular processes [33–35]. PTEN modulates the PI3K/AKT signalling pathway [36, 37]. Pingping Xue and colleagues demonstrated that PTEN which was induced by LPS inhibited the trophoblast invasion via AP-1/NF-κB pathway inhibits trophoblast invasion [38]. However, the regulation of PTEN remains largely unclear. This study demonstrated that PTEN is regulated by LPS-induced MEG3, which absorbs miR-93-5p, thus supplying a novel regulatory mechanism of PTEN.

## Conclusions

This study first introduced LPS for treatment of Leydig cells to simulate Leydig cells in the orchitis environment. Additionally, this study demonstrated that MEG3 expression was increased in Leydig cells under LPS and that silencing of MEG3 attenuated the role of LPS in Leydig cells. Finally, this study revealed that MEG3 exerted its function via modulation of the miR-93-5p/PTEN pathway in Leydig cells under LPS, thus indicating the potential of this signalling pathway in treatment of orchitis.

## Data Availability

The data underlying this article will be shared on reasonable request to the corresponding author.

## Abbreviations

MEG3: Maternally expressed gene 3
LPS: Lipopolysaccharides
NcRNAs: Non-coding RNAs
LncRNAs: Long ncRNAs
DMEM: Dulbecco’s modified Eagle medium
FBS: Foetal bovine serum
siRNAs: Small interfering RNA
RIPA: Radio immunoprecipitation assay
SDS-PAGE: Sodium dodecyl sulfate-polyacrylamide gel electrophoresis
PVDF: Polyvinylidene fluoride
ECL: Chemiluminescence
CCK-8: Cell counting kit-8
EdU: 5-ethynyl-2′-deoxyuridine
RIP: RNA immunoprecipitation
ELISA: Enzyme-linked immunosorbent assay
